# Hypotony maculopathy and choroidal detachment from repeated nocturnal ocular compression in a pediatric patient

**DOI:** 10.1016/j.ajoc.2022.101680

**Published:** 2022-08-12

**Authors:** Elizabeth Ditch, Jeffrey Bloom, Michael Ellis, Robert A. Sisk

**Affiliations:** a Abrahamson Pediatric Eye Institute, Cincinnati Children's Hospital Medical Center, Cincinnati, OH, USA; b Kettering Health, Dayton, OH, USA; c Cincinnati Eye Institute, Cincinnati, OH, USA; d University of Cincinnati Department of Ophthalmology, Cincinnati, OH, USA

**Keywords:** Choroidal detachment, Hypotony, Ocular compression, Ocular ultrasonography, Retinopathy of prematurity

## Abstract

**Purpose:**

To report clinical outcomes of a pediatric patient with unilateral reversible vision loss secondary to hypotony from repeated accidental nocturnal ocular compression from circumaural headphone wear.

**Observations:**

A 17-year-old male with pathologic myopia and history of retinopathy of prematurity previously treated with laser ablation in both eyes presented with reduced visual acuity in his right eye from choroidal detachment and hypotony maculopathy. In the absence of uveitis and intraocular pressure lowering medications, it was determined that repeated nocturnal ocular compression from circumaural headphones created episodes of hypotony. With avoidance of this behavior and in the absence of pathologic aqueous dynamics, intraocular pressure normalized with gradual resolution of choroidal thickening and restoration to baseline visual acuity.

**Conclusions and Importance:**

Persistent and prolonged ocular compression, even unintentionally, can create hypotony with risk for vision loss, maculopathy, and choroidal detachment.

## 1 Introduction

The choroid is one of the most vascular tissues in the body. Therefore, physiological mechanisms such as vortex vein drainage, osmotic pressure, hydrostatic forces, and scleral vessels exist to maintain balance in protein and fluid levels.^1^, ^2^, ^3^ When this equilibrium is disrupted, the blood vessels of the choriocapillaris become enlarged and hyperpermeable causing transudate and exudate to leak into the suprachoroidal space.^1^, ^2^, ^3^, ^4^, ^5^ The process is exacerbated by ciliary body thickening, decreased aqueous production, and hypotony.^3^^,^^4^ When arteriolar and venous pressure exceed the intraocular pressure (IOP), it favors additional leakage of fluid. In patients with pathologic myopia, the compromised retinal and choroidal tissues hamper the choroidal vasculature from responding to changes in IOP.^4^

Ocular hypotony is described as IOP that measures three standard deviations below the mean (less than 6.5 mm Hg) or clinically as an IOP that is low enough to compromise vision.^6^ Although hypotony from glaucoma surgery is the most common cause of choroidal detachment, it can also be caused by trauma, inflammation, infection, scleritis, or idiopathic etiologies.^1^^,^^2^^,^^7^ However, any event which causes a sudden drop in IOP increases the likelihood of choroidal detachment, especially with persistent hypotony, due to dilation of the choroidal vessels that results in leakage.^1^^,^^3^^,^^4^

In this report, we describe a case of repeated unintentional nocturnal ocular compression that resulted in hypotony maculopathy and choroidal detachment.

## 2 Case report

**Fig. 1:**
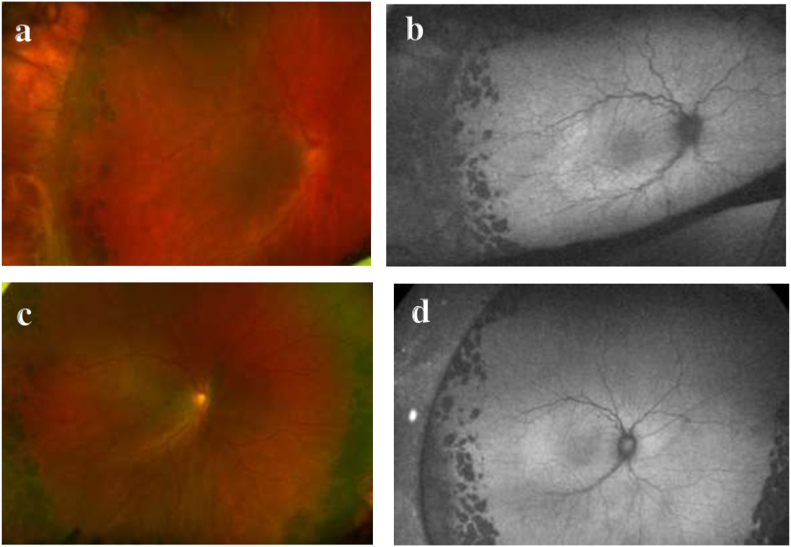
a) Ultra-widefield color fundus photography of the right eye (Optos California®, Marlborough, MA) demonstrating optic disc edema and circumferential choroidal folds towards shallow superotemporal choroidal detachment. The peripheral avascular retina had been treated with laser and cryoablation. b) Ultra-widefield fundus autofluorescence image better demonstrates the striae. c) One week later, the retinal appearance returned to baseline with resolution of the optic disc edema and choroidal folds. d) Corresponding fundus autofluorescence image shows resolution of the choroidal folds. (For interpretation of the references to color in this figure legend, the reader is referred to the Web version of this article.)

**Fig. 2:**
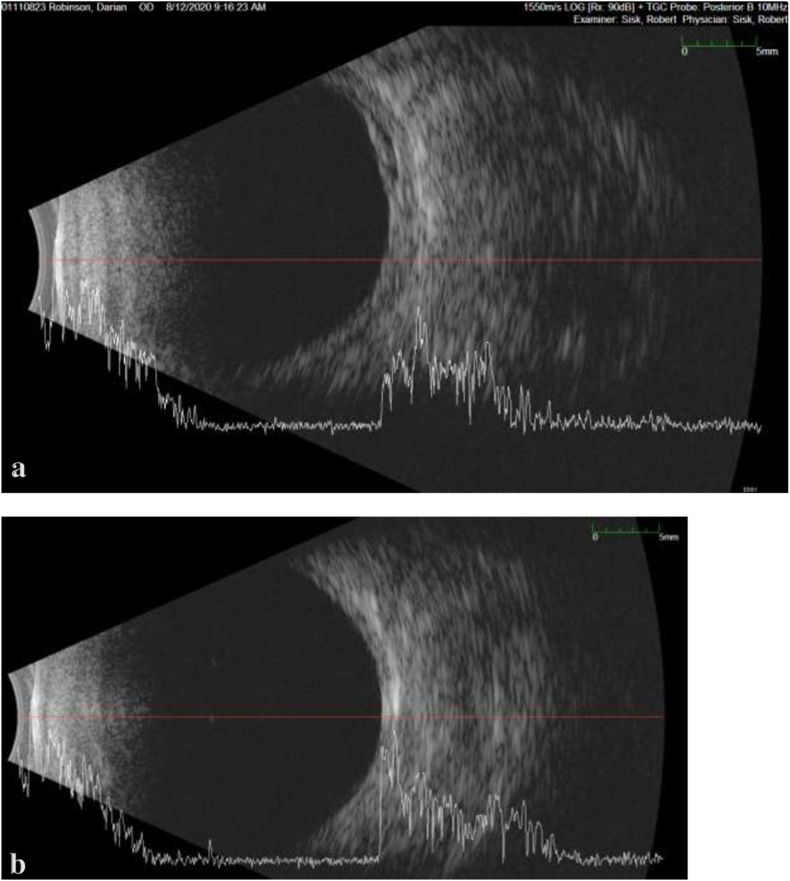
B scan ultrasonography. a) fluid in the suprachoroidal space of the right eye indicating a choroidal detachment, choroidal thickness measured 2.3 mm. b) unremarkable left eye with choroidal thickness of 1.04 mm.

**Fig. 3:**
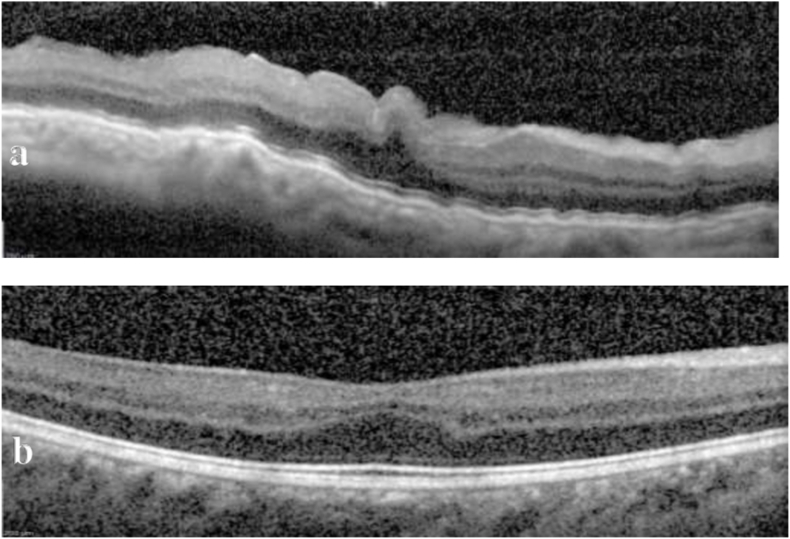
a) Foveal horizontal OCT raster scan demonstrating choroidal and RPE undulation and thickening without subretinal fluid or traction. b) One week later, a similar raster scan showed resolution of choroidal congestion. Subfoveal choroidal thickness improved from 590 μm to 272 μm. The inner retinal layers were not displaced from the fovea at baseline or upon follow-up due to the prior retinopathy of prematurity.

A 17-year-old African American male with pathologic myopia OU (approximately −14D spherical equivalent OU), history of laser ablation for retinopathy of prematurity, and amblyopia OS (baseline 20/200 visual acuity) presented for an urgent evaluation for intermittent aching pain and blurry vision OD that had started two days prior. Best corrected distance visual acuity OD had declined from 20/25 to 20/200 OD. His IOP was 10 mm Hg OD and 15 mm Hg OS by Tonopen®. Slit lamp examination OD revealed moderate conjunctival injection with episcleral dilation, a clear cornea without edema, and a deep anterior chamber with mild cellular reaction. Dilated fundus examination revealed optic disc edema, choroidal engorgement, macular edema, macular choroidal folds OD and superotemporal shallow choroidal detachment OD (Fig. 1). The choroidal thickness measured 2.3 mm OD and 1.04 mm OS with B-scan ultrasonography (Fig. 2). The B-scan also displayed fluid in the suprachoroidal space indicative of a choroidal detachment due to recent hypotony. Macular optical coherence tomography (OCT) demonstrated choroidal and RPE undulation and thickening without subretinal fluid or traction OD (Fig. 3) and normal retinal thickness and contour OS.

Laboratory investigation for causes of uveitis was negative, and review of systems was unremarkable. The patient denied trauma or self-injury and had no prior history of glaucoma, hypotony, or treatments for either problem. The patient's mother volunteered that he frequently lay in bed with bulky circumaural headphones that she witnessed shifted onto his eye while he was asleep. In addition to not wearing headphones to bed, reduced physical activity, and avoiding eye rubbing, treatment consisted of topical prednisolone acetate 1% four times per day and topical atropine 1% twice daily OD. One week later, visual acuity OD improved back to his baseline 20/25 and IOP increased to 18 mm Hg OD. Retinal features returned to baseline.

## 3 Discussion

Ocular compression is utilized clinically to reduce IOP or posterior segment pressure in preparation for intravitreal injections and cataract surgery, or to reduce postoperative IOP elevation following trabeculectomy surgery.^8^^,^^9^ However, in uncontrolled situations like ocular trauma, the consequences of ocular compression and resultant hypotony are more significant: hypotony maculopathy, choroidal detachment, and even retinal detachment. Persistent gouging and excessive eye rubbing are repetitive self-injurious behaviors among some patients with autism, intellectual deficiency, or developmental delay due to triggers such as stress, pain, illness, or frustration from an inability to adequately communicate or complete self-care tasks.^9^

In our case, hypotony likely occurred from repeated nocturnal ocular compression unintentionally which interfered with the typical outflow of the choroidal vascular bed. Disruption of the hydrostatic and osmotic pressure gradients that typically maintain the IOP caused an imbalance in fluid levels leading to choroidal thickening and ciliary body edema.^1^^,^^4^^,^^7^ Aqueous humor production decreased due to a swollen ciliary body causing profound hypotony which ultimately further congested the highly vascular choroid and resulted in a choroidal detachment.^3^, ^4^, ^5^

The roles of steroids to treat choroidal detachments secondary to hypotony are numerous. Steroids are primarily used for their anti-inflammatory effect which reduces leakage from choroidal vessels.^1^^,^^3^ They also aid in resorption of the choroidal detachment to restore normal functioning of the ciliary body and thus restore normal IOP.^3^^,^^5^ Topical steroids are usually initiated first since conservative treatment maximizes visual outcomes, but oral steroids or sub-tenon's triamcinolone acetonide may be necessary if the choroidal detachment is not improving.^1^ Topical cycloplegia may also be used to stabilize the ciliary body and deepen the anterior chamber and resist changes in the lens-iris diaphragm.

## 4 Conclusion

Repeated ocular compression, intentional or otherwise, can produce reversible hypotony with complicating features of maculopathy and choroidal detachment. Limiting self-injurious behavior is essential to prevent the associated vision threatening consequences.

## Patient consent

Consent to publish the case report was not obtained. This report does not contain any personal information that could lead to the identification of the patient.
